# The Frequency of Neurological Symptoms in COVID-19 Patients at a Tertiary Care Hospital in Pakistan

**DOI:** 10.7759/cureus.10360

**Published:** 2020-09-10

**Authors:** Aamir Makda, Sagar Kumar, Ajay Kumar, Vinesh Kumar, Amber Rizwan

**Affiliations:** 1 Neurology, Jinnah Sindh Medical University, Karachi, PAK; 2 Internal Medicine, Ziauddin University, Karachi, PAK; 3 Medicine, Jinnah Post Graduate Medical Center, Karachi, PAK; 4 Internal Medicine, Jinnah Sindh Medical University, Karachi, PAK; 5 Internal Medicine, Ghulam Mohammad Mahar Medical College, Sukkur, PAK; 6 Family Medicine, Jinnah Post Graduate Medical Center, Karachi, PAK

**Keywords:** neurological manifestations, covid-19, pakistan

## Abstract

Introduction

Coronavirus disease 2019 (COVID-19) primarily affects the pulmonary system and presents itself as shortness of breath, fever, and cough. However, it may affect other systems as well, including the nervous system. This study aimed to determine the frequency of neurological symptoms in COVID-19 patients and its association with the severity of the disease.

Methods

This cross-sectional study was conducted at a public sector tertiary care teaching hospital in Karachi, Pakistan, from April to July 2020. All patients with positive polymerase chain reaction (PCR) tests were included, except those with pre-existing neurological and psychiatric conditions.

Results

The most common neurological symptom was dizziness (17.5%), followed by headache (15.7%). Three (2.6%) patients had a stroke. Nine (7.8%) participants had a taste impairment, and another nine (7.8%) had a smell impairment. There was no significant difference in the frequency of neurological symptoms when severe and non-severe disease was compared.

Conclusion

Neurological symptoms are frequent in COVID-19. Care should be taken to identify them early. COVID-19 should be suspected in patients presenting with neurological abnormalities and should be included in the differential diagnosis to prevent further virus transmission.

## Introduction

Coronavirus disease 2019 (COVID-19) is an infectious disease caused by a new strain of coronavirus; its first case was reported in December 2019 in Wuhan, China [1]. The World Health Organization (WHO) declared COVID-19 a worldwide pandemic on March 11, 2020 [2]. COVID-19 most commonly affects the pulmonary system. The most commonly reported symptoms are shortness of breath, fever, and cough. COVID-19 may affect other systems as well. Multiple studies have suggested that COVID-19 may affect both the central and peripheral nervous systems, which have resulted in various neurological manifestations such as headaches, dizziness, cerebrovascular events, altered sense of smell, altered sense of taste, and seizures [3,4]. Mao et al. have reported that one-third of patients with COVID-19 have neurologic symptoms and these symptoms were more common in patients with severe infection [4].

Angiotensin-converting enzyme 2 (ACE2) was identified as the functional receptor for severe acute respiratory syndrome coronavirus 2 (SARS-CoV-2) in January 2020. ACE2 receptors are present in various organs, including the nervous system [5]. The expression and distribution of the ACE2 receptors may be responsible for neurological symptoms in COVID-19 through direct or indirect mechanisms [6].

This study will try to determine the frequency of neurological manifestations in COVID-19 and its association with COVID-19 disease severity. The study aims to help in the diagnosis of COVID-19 patients presenting with non-conventional symptoms.

## Materials and methods

This cross-sectional study was conducted at a public sector tertiary care teaching hospital in Karachi, Pakistan, from April to July 2020. All patients with positive oropharyngeal or nasopharyngeal swabs polymerase chain reaction (PCR) tests for SARS-CoV-2 were included. Patients with prior neurological and psychiatric diseases were excluded from the study. The hospital's ethics committee approved the study protocol, and all patients provided oral informed consent.

A self-structured survey was designed for COVID-19 patients, which recorded various neurological manifestations such as headache, altered sense of smell, altered sense of taste, nausea, dizziness, seizures, cerebrovascular event, and skeletal muscle injury. Acute cerebrovascular disease was diagnosed with the help of clinical symptoms and head CT. Clinical symptoms were used to diagnose seizures. Skeletal muscle injury was diagnosed with the help of laboratory values (serum kinase level greater than 200 U/L) and skeletal muscle pain [7]. Pakistan's national guidelines for the management of COVID-19 were used to label patients with severe disease [8].

Data were processed and analyzed using SPSS Statistics for Windows, version 22.0 (IBM Corp., Armonk, NY). Quantitative variables were summarized as mean and standard deviation (SD), and qualitative variables as frequency and percentage. Qualitative variables were cross-tabulated with disease severity and compared with chi-square tests. A p-value of less than 0.05 indicated that the difference in neurological manifestation between mild to moderate and severe disease was significant enough to discard the null hypothesis.

## Results

A total of 114 patients was selected for the study. The mean age of the participants was 51 ± 14 years. There were 49 (42.9%) participants who were less than 50 years of age and 65 (57.1%) participants older than 50 years. There were 52 (45.6%) females and 62 (54.4%) males. There was no significant difference in severity when patients were stratified for age and gender; 28 (24.5%) patients had neurological manifestations. The most common neurological symptom was dizziness (17.5%) followed by headache (15.7%). Nine (7.8%) participants had taste impairment, and another nine (7.8%) had a smell impairment. There was no significant difference in the frequency of neurological symptoms when severe and non-severe disease was compared (Table 1).

**Table 1:** Frequency of neurological manifestations *P-value was calculated by comparing severe and non-severe groups. ^a^Significant result. ^b^Non-significant result SD: standard deviation

Neurological manifestations	Total (n=114)	Severe (n=62)	Non-severe (n=52)	P-value*
Age in years, mean (SD)	51 (14)	58 (15)	46 (14)	0.00^a^
Age in years, n (%)				
<50	49 (42.9%)	29 (46.8%)	28 (53.4%)	0.56^b^
≥50	65 (57.1%)	33 (53.2%)	24 (46.6%)	
Sex, n (%)				
Female	52 (45.6%)	30 (48.38%)	22 (42.3%)	0.51^b^
Male	62 (54.4%)	32 (51.6%)	30 (57.6%)	
Nervous system symptoms, n (%)				
Dizziness	20 (17.5%)	11 (17.7%)	9 (17.3%)	0.95^b^
Headache	18 (15.7%)	10 (16.1%)	8 (15.3%)	0.91^b^
Impaired consciousness	10 (8.7%)	8 (12.9%)	2 (3.8%)	0.08^b^
Acute cerebrovascular disease	3 (2.6%)	3 (4.8%)	0	0.24^b^
Seizure	1 (0.9%)	1 (1.6%)	0	1.00^b^
Impairment, n (%)				
Taste	9 (7.8%)	4 (6.4%)	5 (9.6%)	0.38^b^
Smell	9 (7.8%)	5 (8.0%)	4 (7.6%)	0.94^b^
Vision	2 (1.7%)	1 (1.6%)	1 (1.6%)	0.90^b^
Nerve pain, n (%)	4 (3.5%)	2 (3.2%)	2 (3.8%)	0.85^b^
Skeletal muscle injury, n (%)	12 (10.5%)	9 (14.4%)	3 (5.7%)	0.12^b^

## Discussion

SARS‐CoV‐2 may disturb the central nervous system through various mechanisms, including direct invasion, hypoxia, hypercoagulopathy, and immune‐mediated injury [9]. In our study, 24.5% had neurological symptoms. Another study from Pakistan showed that 18.5% of patients with COVID-19 had neurological symptoms [10]. A study from Wuhan showed a much higher frequency (38%) of neurological symptoms [4]. Our study showed that dizziness (17.5%), headache (15.7%), skeletal muscle injury (10.5%), and impaired consciousness (8.7%) were the most common neurological symptoms.

Dizziness was the most commonly presented neurological symptom in our study. Jacky Sia, in his study, stated that dizziness could be the sole clinical manifestation of COVID-19 infection [11]. Headache is also a common neurological presentation in COVID-19. Various studies have reported headaches to be as common as 6.5-53% [12]. Headache was a common presentation in severe acute respiratory syndrome (SARS), another disease caused by coronavirus [13].

Acute cerebrovascular disease (stroke) was present in three patients in our cohort. Various factors are responsible for stroke in patients with COVID-19. Viral infection of endothelial cells, which may damage blood vessels, may increase the risk of stroke. Viral infection of vascular endothelial cells accompanied by damage to the vasculature can predispose to infarct [14,15]. SARS-CoV-2 is associated with increased cytokines, which may damage neurons and thereby lead to stroke [16,17]. Another factor is hypercoagulability caused by SARS-CoV-2 itself, which may cause stroke without any predisposing factors [18,19].

In this study, 12 (10.5%) participants had skeletal muscle injury. Muscle injury has been reported by various studies [4,10]. Muscle injury may result in an increase in creatine kinase, alanine, and aspartate transferase. Some studies have reported rhabdomyolysis in COVID-19 patients [20].

In this study, nine (7.8%) participants reported altered taste. One possible mechanism for altered taste is that salivary glands, as they have (ACE2) receptors, may be an early target of SARS-CoV-2 infection. This infection of the salivary gland may decrease saliva flow, which may be responsible for altered taste in COVID-19 [21]. Nine (7.8%) participants complained of altered smell in our study. In some patients with COVID-19, anosmia, or hyposmia is the presenting complaint [22]. It has been observed that patients who present with anosmia are mostly young and asymptomatic [23].

The present study had several limitations. Firstly, all our data were collected from one institute. Also, long-term implications of neurological symptoms were not noted, and not all tests were performed in all participants included in the study as they were self-isolated at home after the initial diagnosis.

Our study, along with other studies, has shown that neurological manifestations are not uncommon in COVID-19. All patients presenting with respiratory and neurological signs and symptoms should be tested for COVID-19 to avoid delay in diagnosing the condition. A multidisciplinary approach is needed to manage COVID-19 patients presenting with neurological symptoms.

## Conclusions

In our study, neurological manifestations among patients were not uncommon. Patients commonly reported dizziness, headache, impaired consciousness, skeletal muscle injury, and altered senses. Patients presenting with neurological manifestation should undergo testing for COVID-19 during this pandemic. Clinicians and researchers should make a joint effort to gather more data related to neurological manifestations in COVID-19 and, if deemed necessary, changes in diagnostic guidelines should be made.
